# Supplementary material for: Biomimetic stimulation patterns drive natural artificial touch percepts using intracortical microstimulation in humans
Source: J Neural Eng. Author manuscript; Available in PMC 2026 Sep 13. (PMC13571258; doi:10.1088/1741-2552/adc2d4)
Supplement: Supplementary file 1 [file NIHMS2206654-supplement-supp_info.pdf]

Supplementary Information

Participant	Type of Touch	Verbal Description While Blindfolded
**P3**	Mechanical indentation on thenar eminence of the right palm (2mm at 10mm/s)	"A poke"
	Light indentation from Q-tip on the thenar eminence of the right palm	"A poke"
	Light indentation from Q-tip on the right side of the face	"A poke. Similar sensation to what I felt on my hand"
**P2**	Mechanical indentation on thenar eminence of the right palm (2mm at 10mm/s)	"Dull pressure poke that went down and up and felt very focal and pin pointy as in one specific location."<br>"It was a 'pressurey' poke, it had a start and stop and felt like being poked."
	Light indentation from Q-tip on the thenar eminence of the right palm	"A poke"<br>"Just a poke"
	Light indentation from Q-tip on the right side of the face	"Felt like a light poke"<br>"Also a poke"
**C1**	Mechanical indentation on thenar eminence of the right palm (2mm at 10mm/s)	"A poke"
	Light indentation from Q-tip on the thumb and thenar eminence of the right palm	"It felt like a Q-tip on, like the thumb palm side upper part of the thumb. Same thing, a little closer to the palm."
	Light indentation from Q-tip on the left side of the face	"On the cheek, Q-tip. Some felt a little more pokey on the check"

Supplementary Table 1. Verbal reports of the perceived sensations resulting from indentation on the hand and face. Participants, who were blindfolded, reported the sensations from mechanical indentations on the thenar eminence on their right hand (same parameters used in this study), and from indentations with a Q-tip on both the thenar eminence on their right hand and the right side of their face (a location unaffected by their injury).

Participant	ICMS Paradigm	Poke	Pressure	Dynamics	Sharp	Quality	Touch	Less Warm	Less Tingle	Focal	Stim Intensity	Poke Intensity	Only Stim Intensity
**P2 (77%)**	S Bio	-	-	-	-	-	-	4	1	-	3	-	-
	S None	4	1	4	-	-	-	12	1	2	2	-	3
	S Lin	4	1	1	**1**	-	-	-	1	3	5	**1**	1
	A Bio	3	**5**	5	1	-	-	9	5	2	**1**	-	-
	A None	4	-	6	2	1	-	2	3	3	-	-	-
	**Total**	**15**	**7**	**16**	**4**	**1**	**-**	**27**	**11**	**10**	**11**	**1**	**4**
**P3 (18%)**	S Bio	**2**	3	1	**2**	1	1	-	-	-	2	-	-
	S None	-	2	4	-	1	1	-	-	-	2	2	1
	**Total**	**2**	**5**	**5**	**2**	**2**	**2**	**-**	**-**	**-**	**4**	**2**	**1**
**C1 (5%)**	**Total**	**-**	**-**	**6**	**1**	**-**	**-**	**-**	**-**	**-**	**-**	**-**	**-**
**Grand Total**		17	12	27	7	3	2	27	11	10	15	3	5
**Percentage of Total (%)**		12	9	19	5	2	1	19	8	7	11	2	4

Supplementary Table 2. Key descriptive terms used in the verbal reports from participants regarding their perceptual experience during the experiment. Participants IDs are listed in the leftmost column, along with the total percentage of descriptors they contributed. Rows are organized by electrode/electrode group performance: simple biomimetic (S Bio), simple at chance (S None), simple linear (S Lin), advanced biomimetic (A Bio), advanced at chance (A None). Table cells are colored if a descriptor was used only for electrodes/electrode groups where biomimetic ICMS was chosen above chance (green) or linear ICMS was chosen above chance (purple).

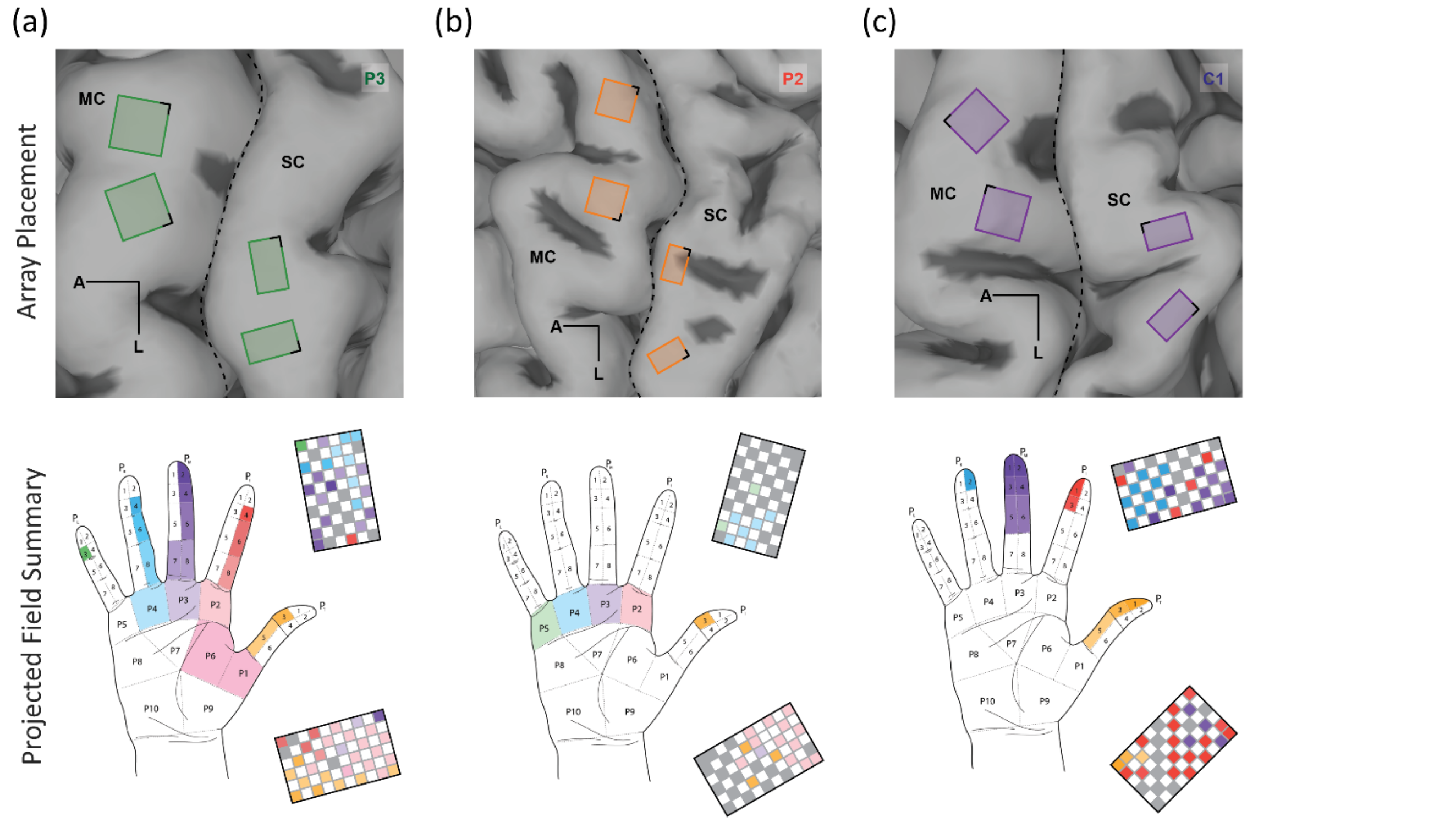

Supplementary Figure 1. Array placement and projected field maps for each participant (a) P3, (b) P2, and (c) C1. (Top row) Array placements in the motor cortex (MC) and somatosensory cortex (SC). (Bottom row) Diagrams representing the distributions of the tactile sensations evoked by ICMS by any electrode on the palmar surface of the hand. Projected fields were combined during the data collection period of this study. The medial array is on top, and the lateral array is on bottom. Each colored electrode evokes a sensation on the correspondingly colored region of the hand.

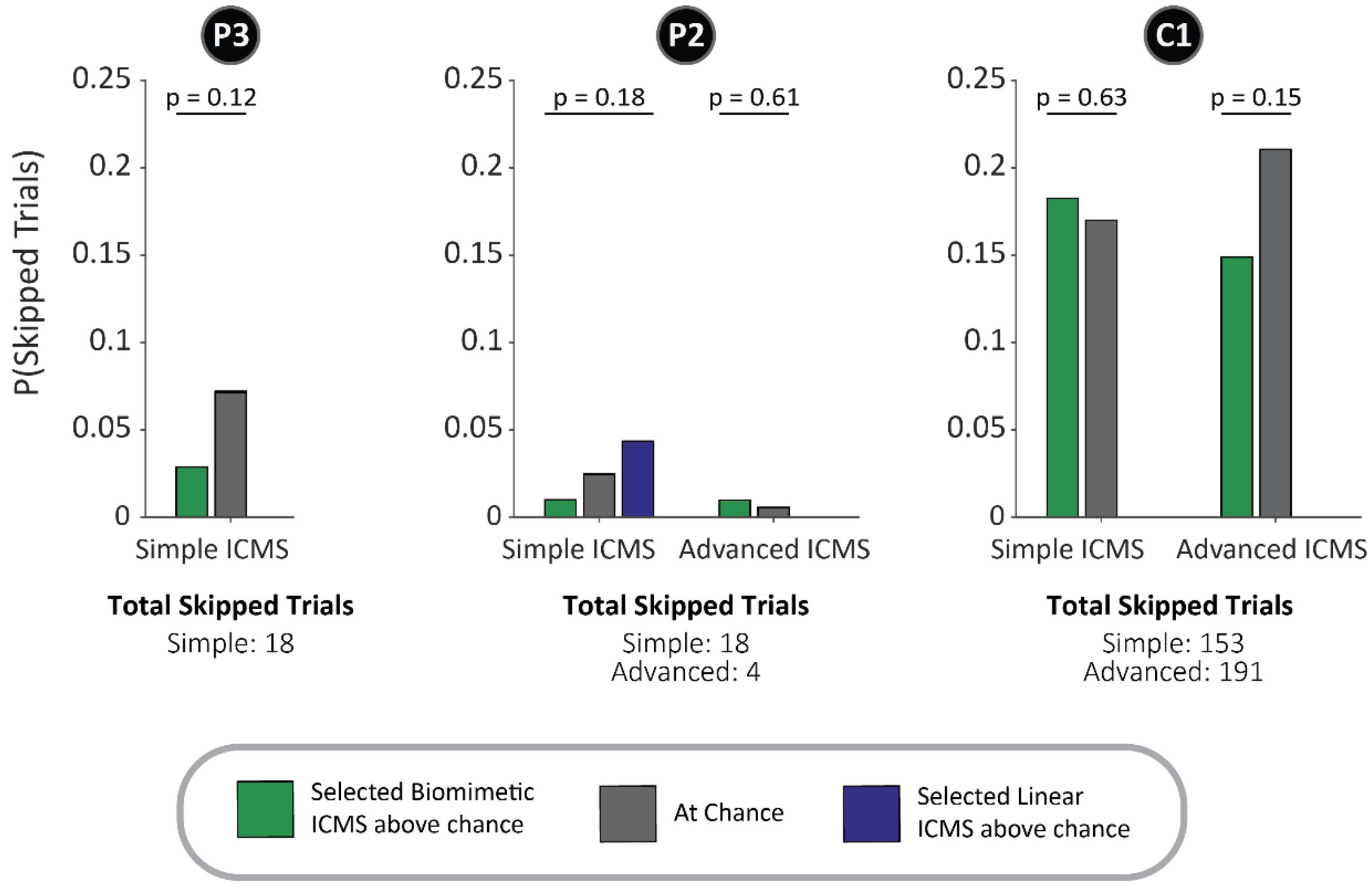

Supplementary Figure 2. The proportion of skipped trials based on electrode/electrode group performance. The bar graph for each participant shows the proportion of times they requested to skip and repeat a trial, split by the ICMS paradigm (simple or advanced) and experimental outcome (green = selected biomimetic ICMS above chance levels, gray = at chance levels, purple = selected linear ICMS above chance levels). There was no significant difference between any of the proportions (Chi-squared test). The total number of skipped trials is shown below each bar graph. Participant identifiers are included in black circles.

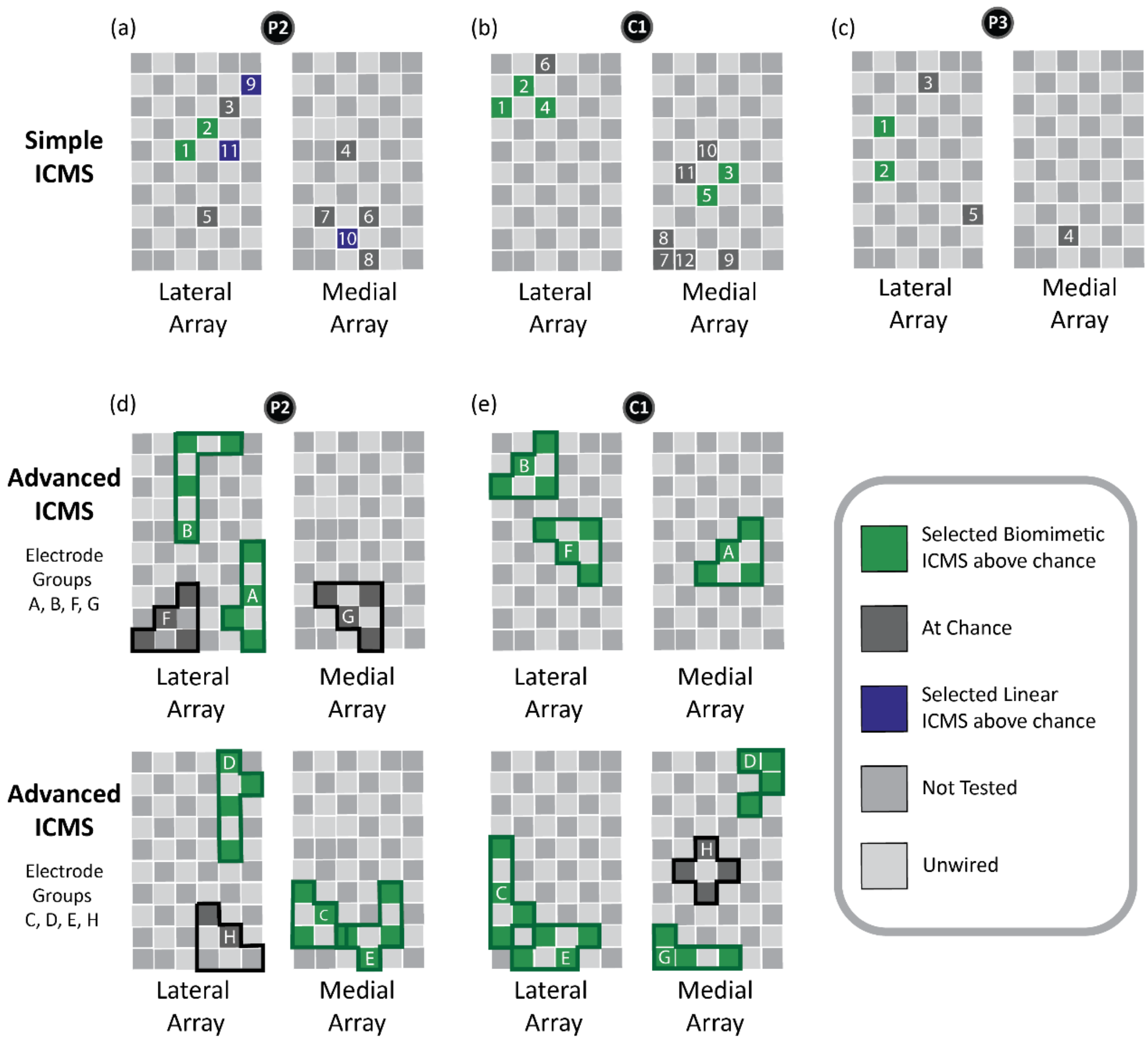

Supplementary Figure 3. Electrode locations were not related to task choices. (a-c) Location of tested electrodes on the two arrays for each participant. Each electrode is color-coded based on whether one of the ICMS encoding schemes was selected as a better perceptual representation of the mechanical stimulus above chance, combined across all testing days (green = biomimetic trials above chance, gray = at chance, purple = linear trials above chance, $p < 0.05$, Chi-square goodness-of-fit test). (d-e) Location of electrode groups on the two arrays for P2 and C1. Arrays are duplicated in a column due to some electrode overlap between stimulation groups. The group letter is located on the electrode that maintained constant stimulation for the entire second. Electrodes are colored as in (a-c). Participant identifiers are included in black circles.

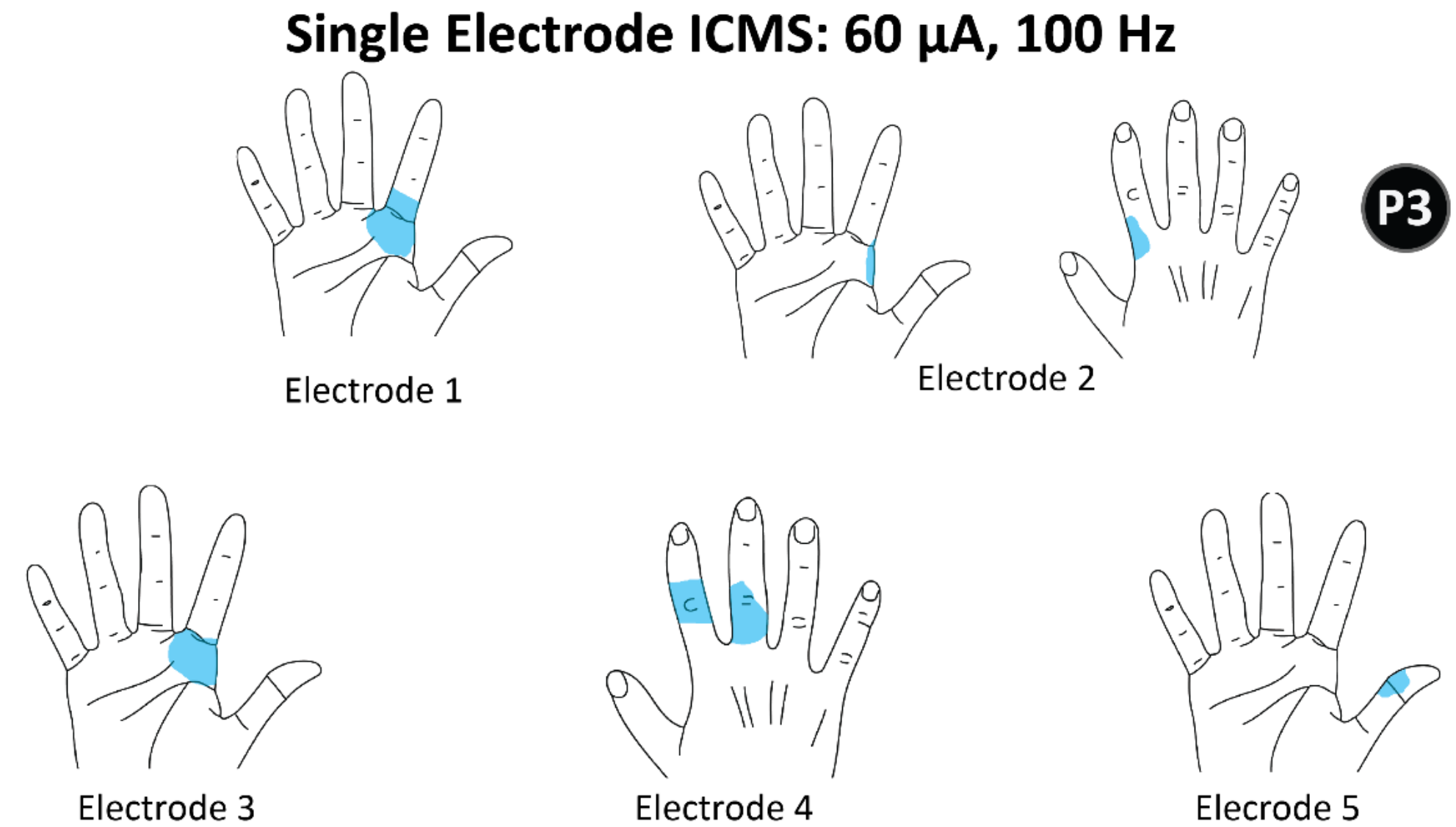

Supplementary Figure 4. Projected fields for each electrode tested with simple ICMS in participant P3. Blue-colored regions indicate the location on the hand where 60 μA, 100 Hz, 1 s ICMS trains were perceived by P3. For clarity, the dorsal or palmar side was excluded if no percepts were reported. The participant identifier is included in the black circle.

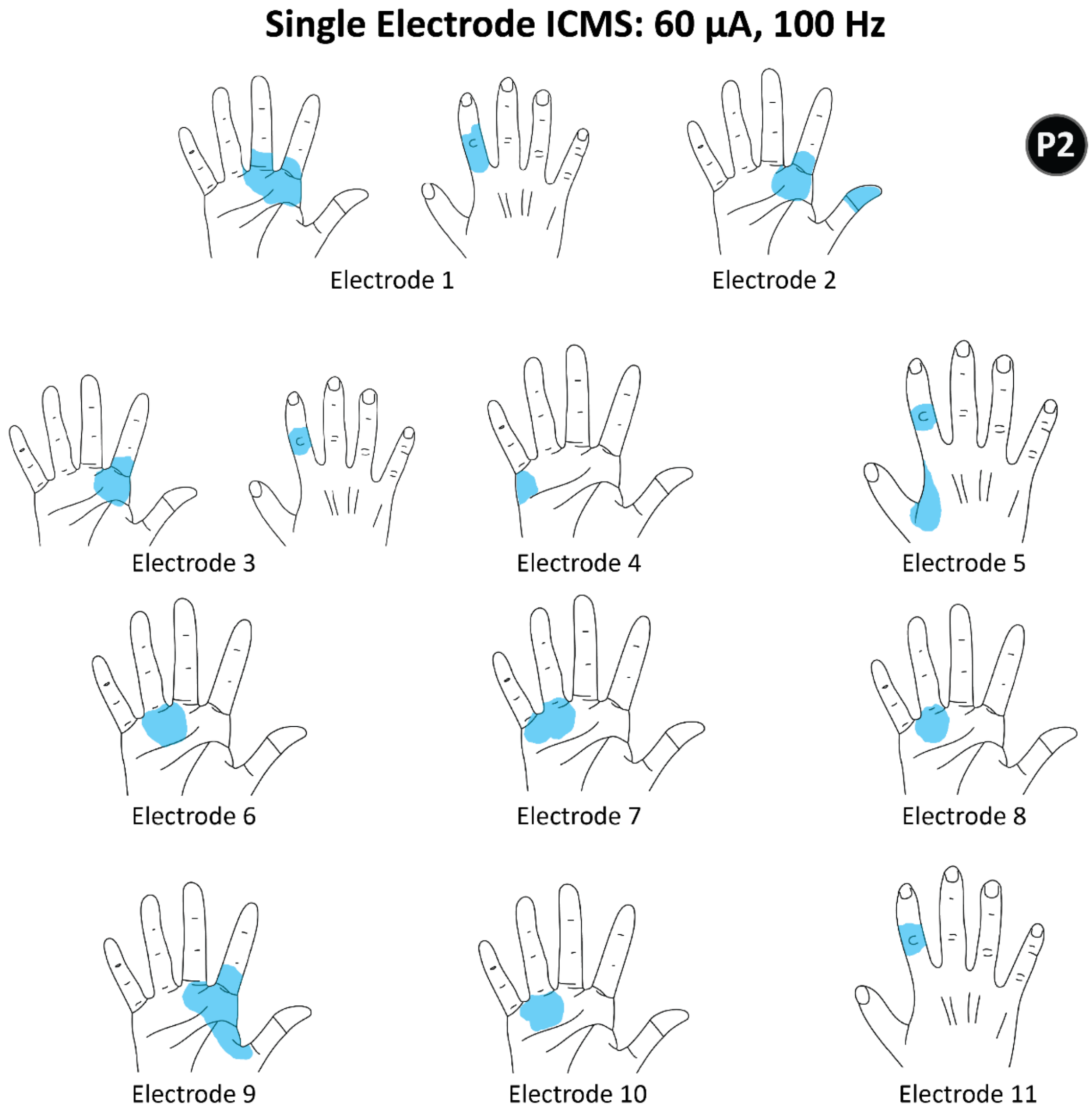

Supplementary Figure 5. Projected fields for each electrode tested with simple ICMS in participant P2. Blue-colored regions indicate the location on the hand where 60 µA, 100 Hz, 1 s ICMS trains were perceived by P2. For clarity, the dorsal or palmar side was excluded if no percepts were reported.

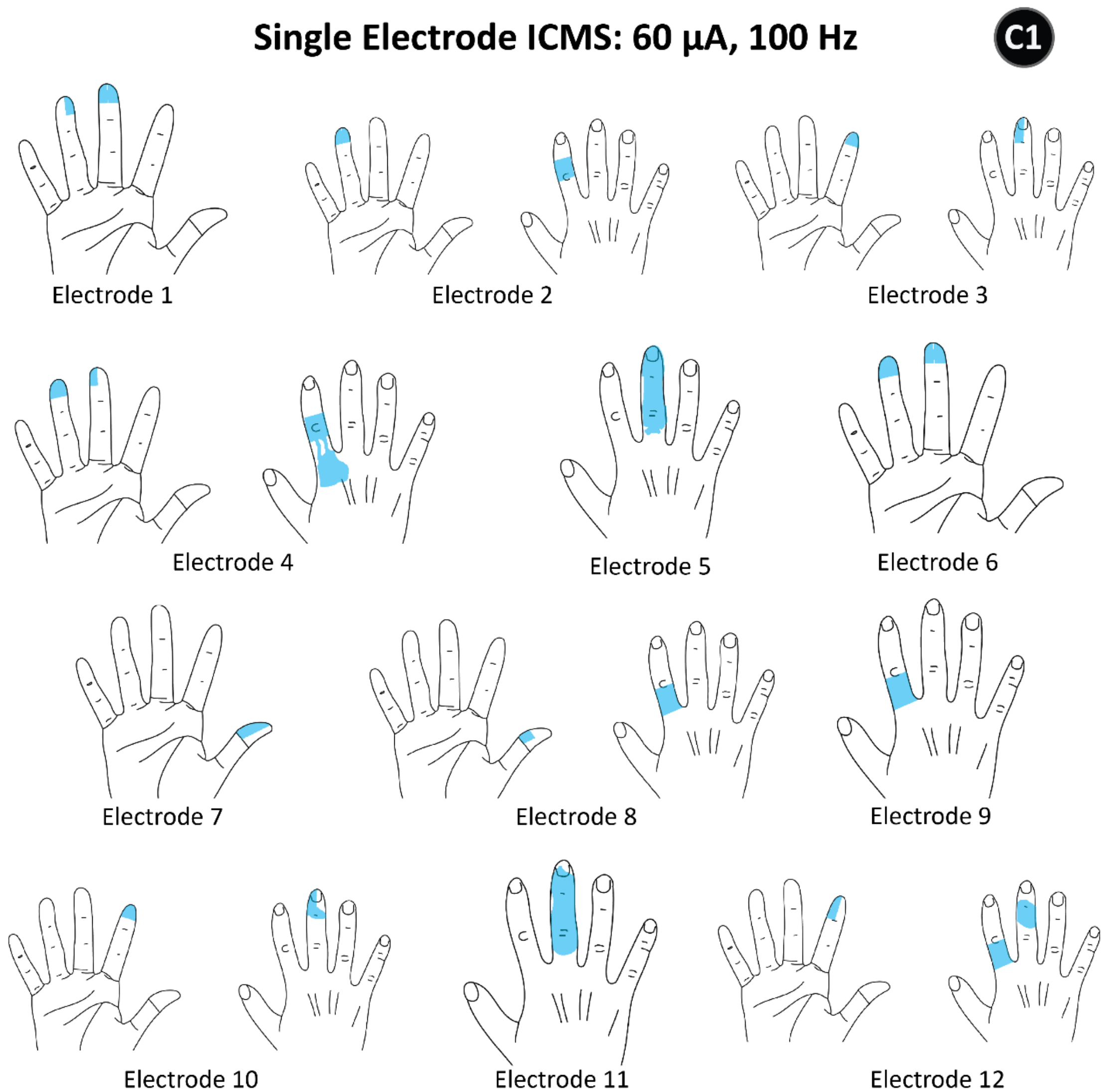

Supplementary Figure 6. Projected fields for each electrode tested with simple ICMS in participant C1. Blue-colored regions indicate the location on the hand where 60 µA, 100 Hz, 1 s ICMS trains were perceived by C1. For clarity, the dorsal or palmar side was excluded if no percepts were reported.

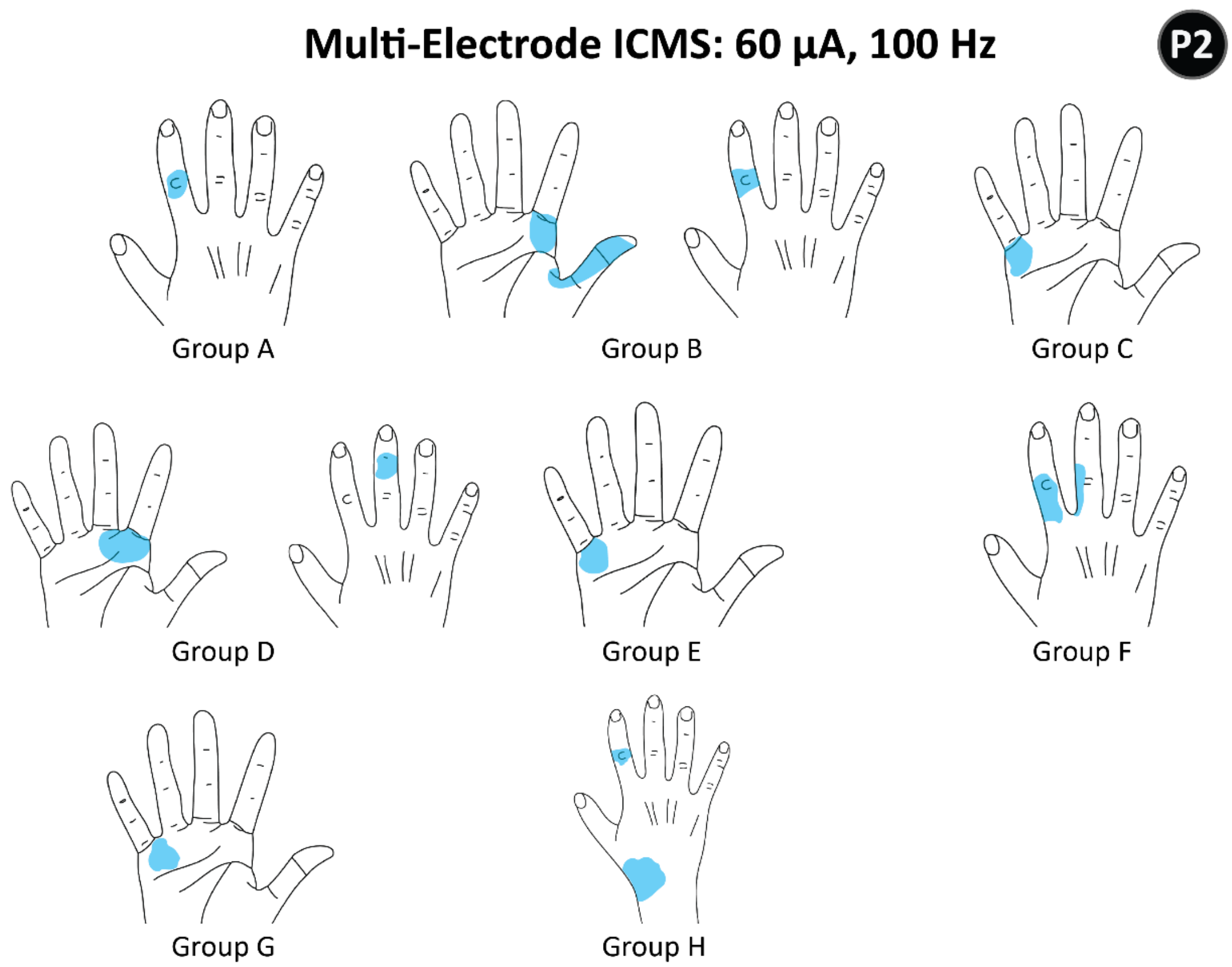

Supplementary Figure 7. Projected fields for each electrode group tested with advanced ICMS in participant P2. Blue-colored regions indicate the location on the hand where 60 μA, 100 Hz, 1 s ICMS trains across all four electrodes were perceived by P2. For clarity, the dorsal or palmar side was excluded if no percepts were reported.

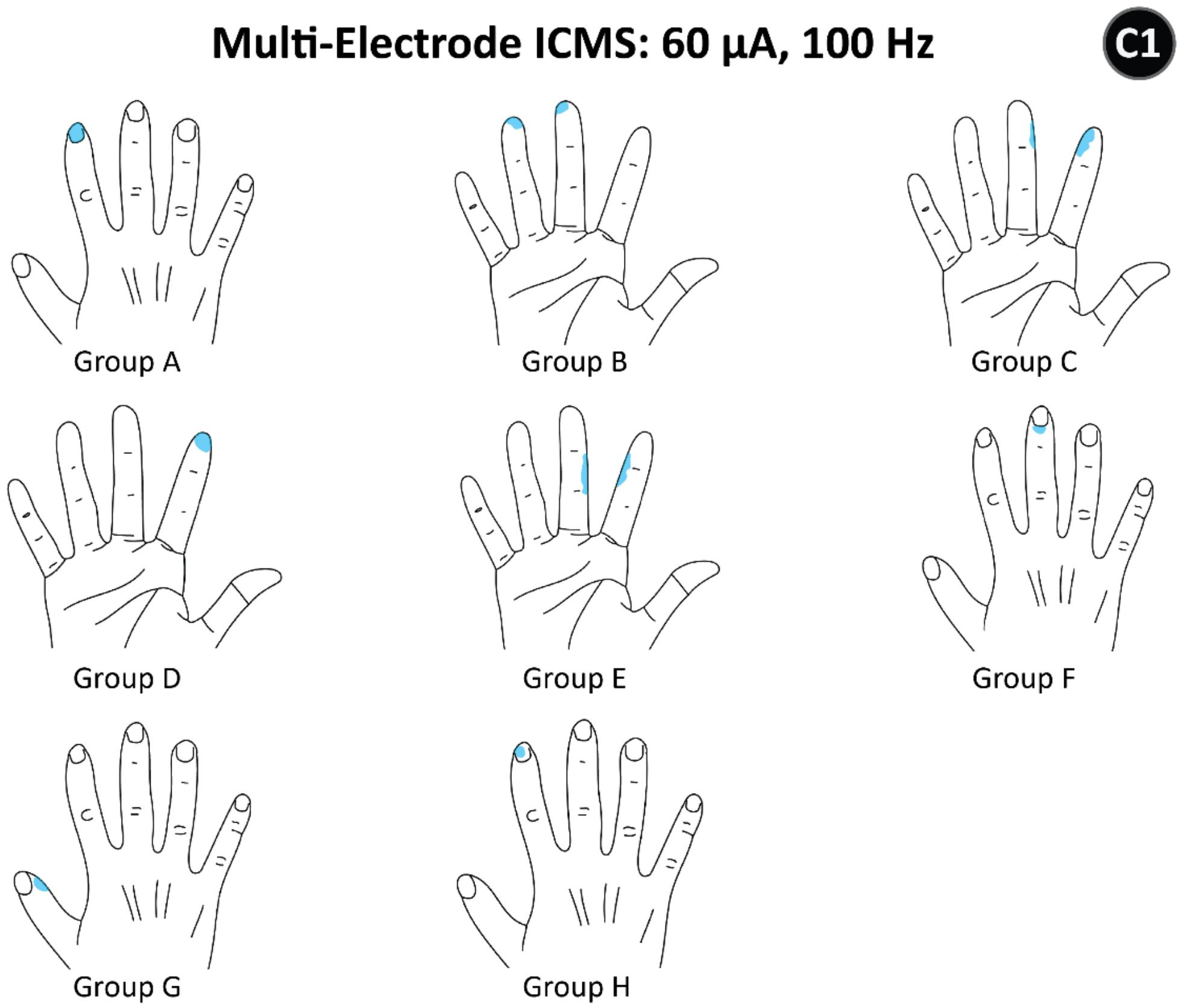

Supplementary Figure 8. Projected fields for each electrode group tested with advanced ICMS in participant C1. Blue-colored regions indicate the location on the hand where 60 µA, 100 Hz, 1 s ICMS trains across all four electrodes were perceived by C1. For clarity, the dorsal or palmar side was excluded if no percepts were reported.

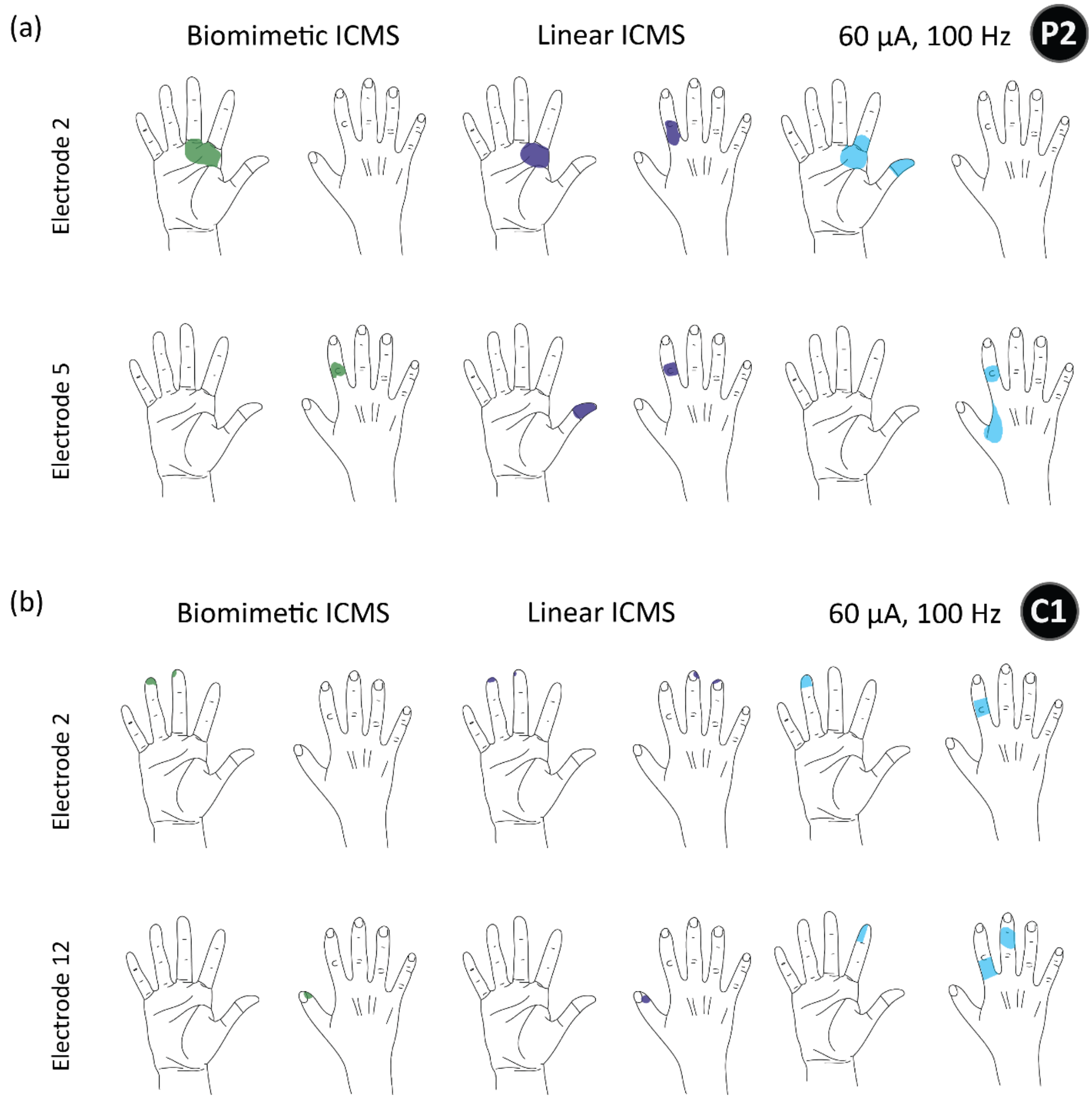

Supplementary Figure 9. Projected fields from simple biomimetic and linear ICMS. The left column displays projected fields collected on one day from simple biomimetic ICMS. The middle column displays projected fields collected on one day from simple linear ICMS. The right column displays combined projected fields over the entire data collection period, using the ICMS parameters (60 µA, 100 Hz) that are standard when collecting projected fields [13,31]. (a) Electrode 2 was an electrode where simple biomimetic ICMS was selected above chance, while electrode 5 was an electrode at chance levels. (b) Electrode 2 was an electrode where simple biomimetic ICMS was selected above chance, while electrode 12 was an electrode at chance levels. Participant identifiers are included in black circles.

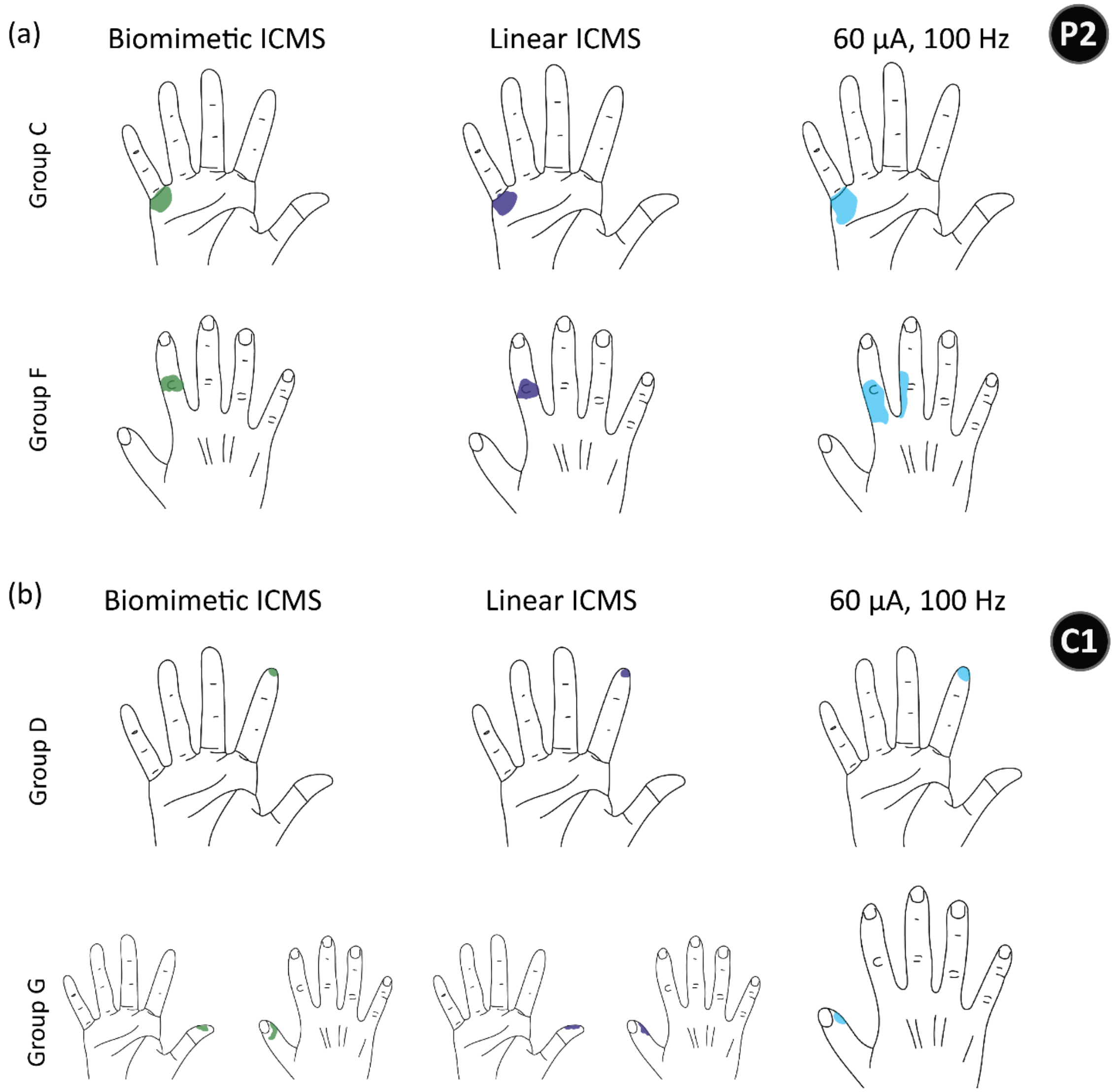

Supplementary Figure 10. Projected fields from advanced biomimetic and linear ICMS. The left column displays projected fields collected on one day from advanced biomimetic ICMS. The middle column displays projected fields collected on one day from advanced linear ICMS. The right column displays combined projected fields over the entire data collection period, using the ICMS parameters (60 µA, 100 Hz) across all four electrodes that are standard when collecting projected fields [13,31]. For clarity, the dorsal or palmer side was not included if no percepts were reported. (a) Group C was an electrode group where advanced biomimetic ICMS was selected above chance, while Group F was an electrode group that was at chance levels. (b) Group D was an electrode group where advanced biomimetic ICMS was selected above chance, while Group G also had advanced biomimetic ICMS being selected above chance, but for a lower proportion of trials compared to Group D.

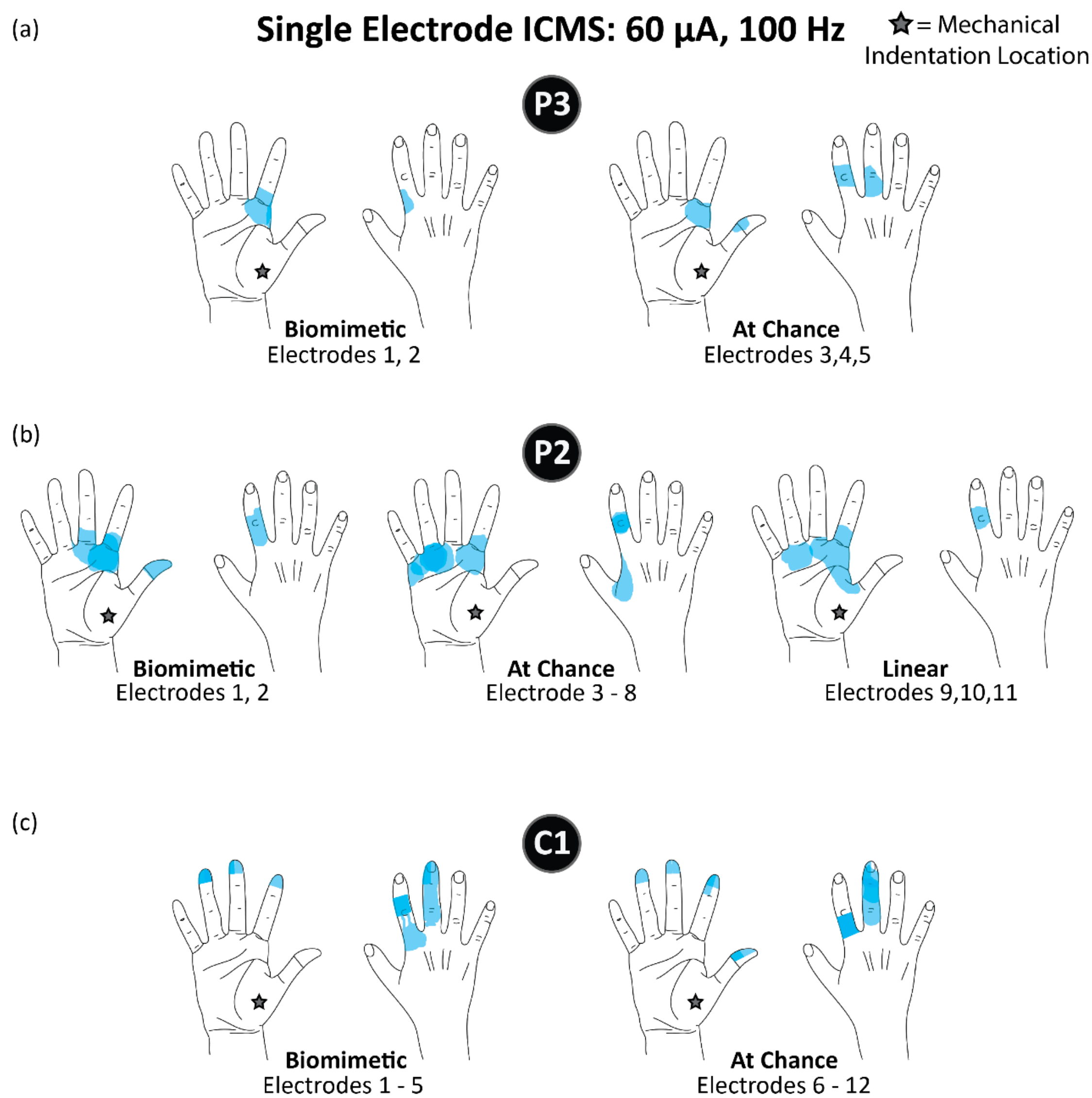

Supplementary Figure 11. Projected field overlap between electrodes that evoked different perceptual choices for simple ICMS encoding schemes. (a-c) Blue-colored regions indicate the location on the hand where 60 µA, 100 Hz, 1 s ICMS trains were perceived by each participant. The star symbol marks the approximate location of the mechanical indentation. Participant identifiers are included in black circles.

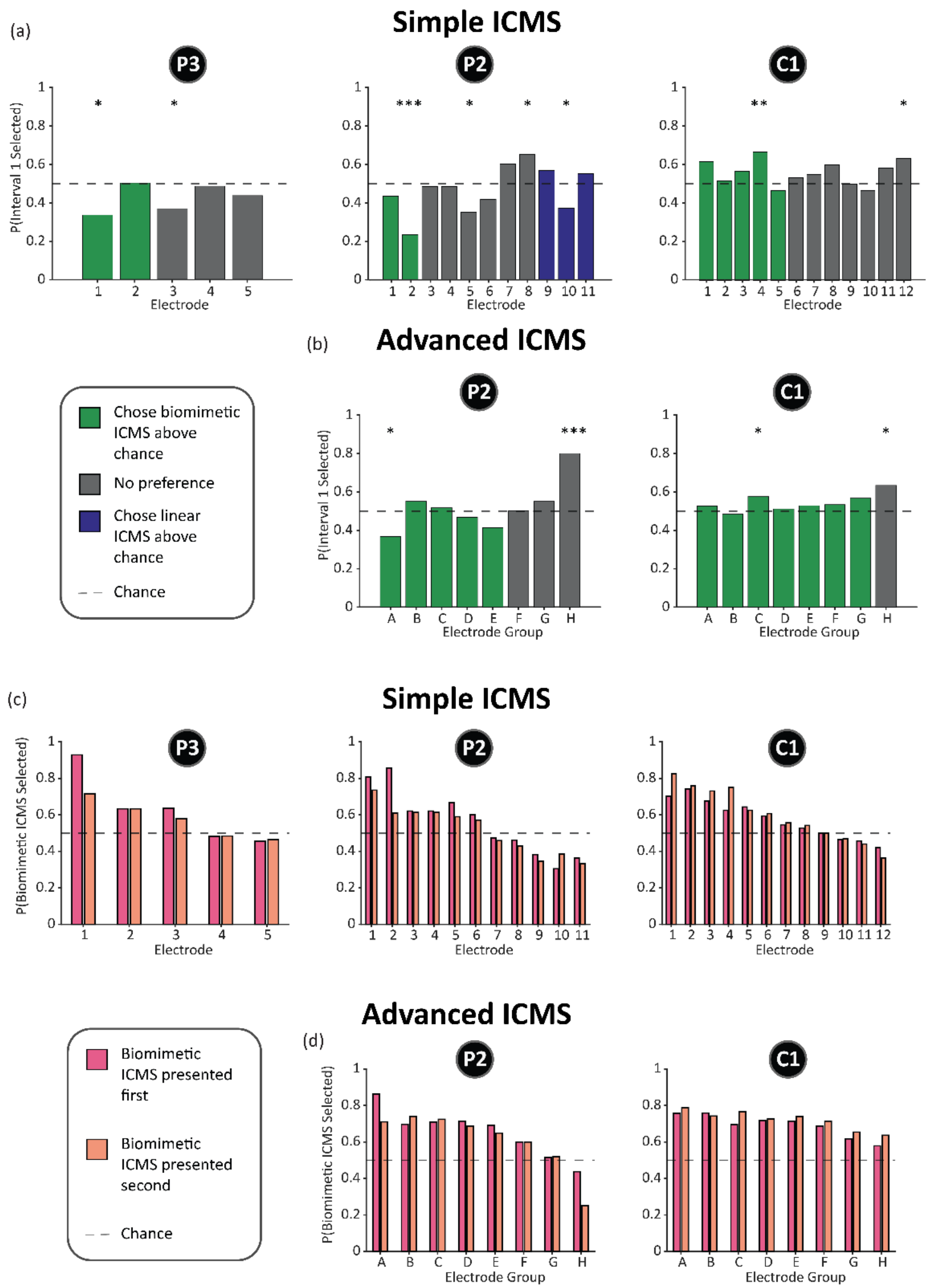

Supplementary Figure 12. Bias and order effects did not impact performance. (a,b) The proportion of trials in which the first interval was selected (***p < 0.001, **p < 0.01, *p < 0.05, Chi-squared goodness-of-fit test). Each color indicates the ICMS encoding scheme that was chosen to best match the mechanical stimulus (green = biomimetic, gray = at chance, purple = linear). (c,d) The proportion of trials where the interval containing biomimetic ICMS was selected, split by whether biomimetic ICMS was presented first (pink) or second (orange). The proportion of trials in which biomimetic ICMS was selected did not differ between if it was presented in the first or second interval for any electrode or electrode group in any participant (p-value range: 0.08 - 1.0, panel (c); p-value range: 0.18 - 1.0, panel (d); Chi-squared test).

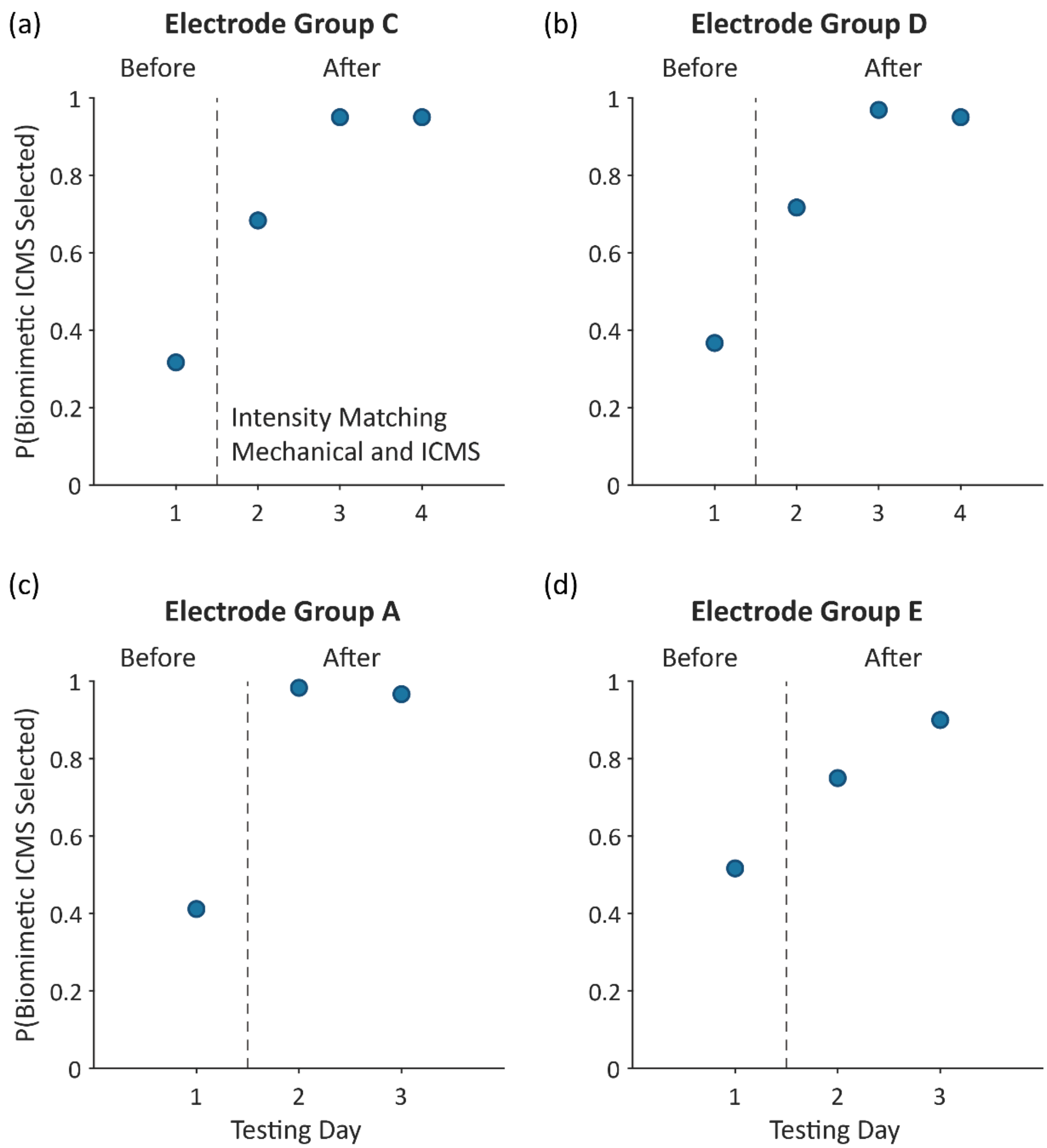

Supplementary Figure 13. Effect of intensity matching method on performance for advanced ICMS in participant C1. (a-d) The proportion of trials where advanced biomimetic ICMS was selected across testing days for four electrode groups in participant C1. The dashed line indicates when intensity matching between mechanical indentation and ICMS was improved.

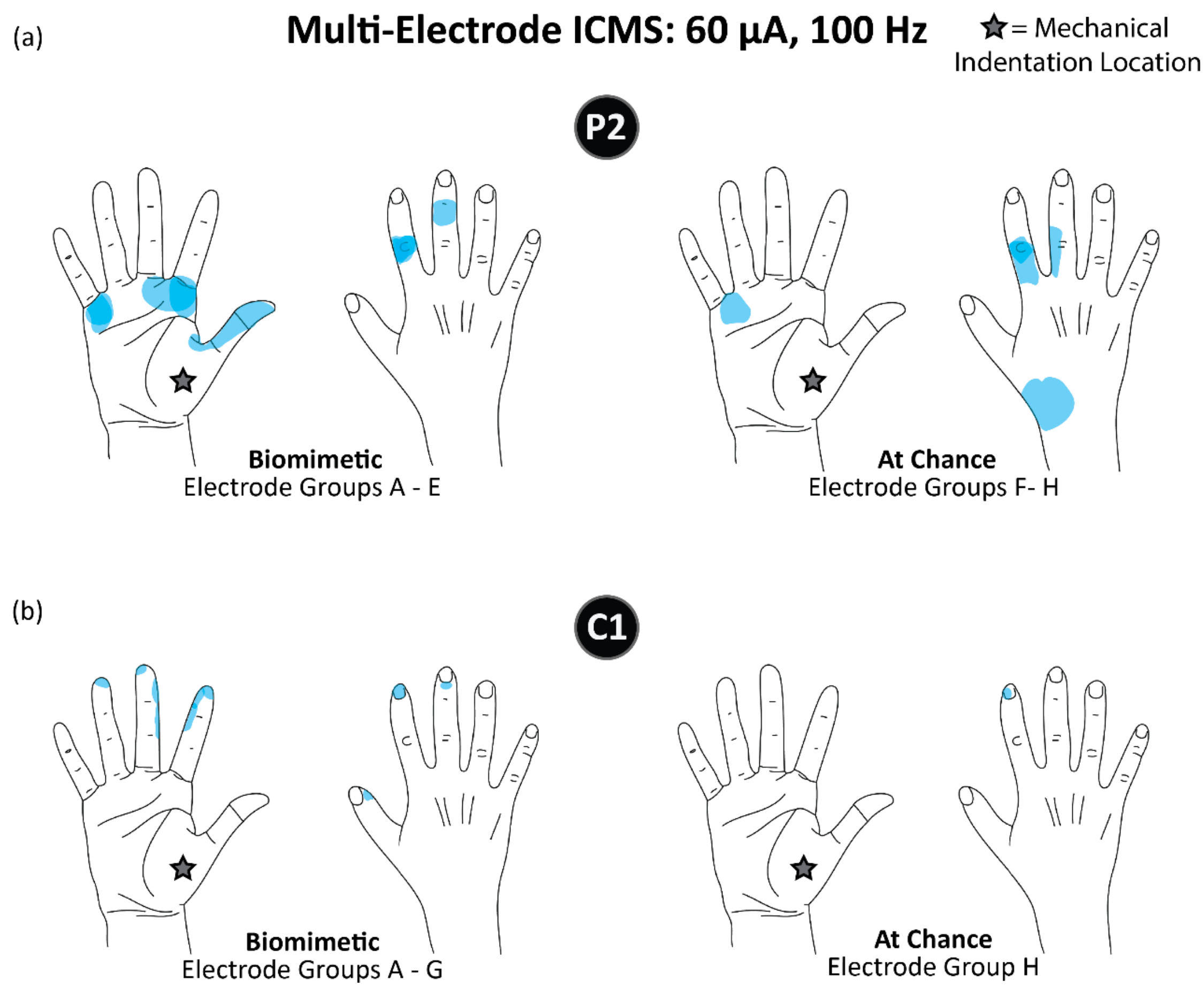

Supplementary Figure 14. Projected field overlap between electrode groups that evoked different perceptual choices for advanced ICMS encoding schemes. (a-c). Blue-colored regions indicate the locations on the hand where 60 µA, 100 Hz, 1 s ICMS trains across a group of four electrodes were perceived by each participant. The star symbol marks the approximate location of the mechanical indentation. Participant identifiers are included in black circles.
